# Clinical and Microbiological Risk Factors for 30-Day Mortality of Bloodstream Infections Caused by OXA-48-Producing *Klebsiella pneumoniae*

**DOI:** 10.3390/pathogens13010011

**Published:** 2023-12-21

**Authors:** Pilar Lumbreras-Iglesias, Edurne Rodrigo-Arrazola, Lucía López-Amor, Jonathan Fernández-Suárez, María Rosario Rodicio, Javier Fernández

**Affiliations:** 1 Traslational Microbiology Group, Health Research Institute of the Principality of Asturias (ISPA), 33011 Oviedo, Spain; uo231178@uniovi.es (P.L.-I.); jhonatan.fernandez@sespa.es (J.F.-S.); 2 Department of Clinical Microbiology, Central University Hospital of Asturias (HUCA), 33011 Oviedo, Spain; edurnearrazola@gmail.com; 3 Hematological Malignancies Group, Health Research Institute of the Principality of Asturias (ISPA), 33011 Oviedo, Spain; 4 Department of Intensive Care Medicine, San Agustín University Hospital (HUSA), 33401 Avilés, Spain; lucia.lopez@sespa.es; 5 Department of Functional Biology, Microbiology Area, University of Oviedo, 33006 Oviedo, Spain; 6 Research & Innovation, Artificial Intelligence and Statistical Department, Pragmatech AI Solutions, 33001 Oviedo, Spain; 7 Biomedical Research Networking Center—Respiratory Diseases, 28029 Madrid, Spain

**Keywords:** *Klebsiella pneumoniae*, bloodstream infection, risk factors, mortality, carbapenem resistance, *bla*_OXA-48_, ST147

## Abstract

Bloodstream infections (BSI) caused by carbapenem-resistant *Klebsiella pneumoniae* are associated with high morbidity and mortality, and the therapy options available for their treatment are frequently scarce. The aim of this study was to analyze risk factors for 30-day mortality in patients with BSI caused by OXA-48-producing *K. pneumoniae*. The clinical and treatment features of the patients, who attended a single hospital over a five-year period, were retrospectively reviewed. The microbiological features, including the sequence types (ST) and the somatic (O) and capsular (K) antigens, as well as their resistance properties, comprising phenotypes and genetic background, were also considered. To identify the risk factors for 30-day mortality, uni- and multivariate statistical analyses were performed. The univariate analysis revealed statistically significant correlations for age, male gender, lower respiratory system infection, infection by ST147 isolates, and infection by isolates expressing the K64 antigen. The multivariate analysis, applied to variables yielding *p*-values close to or lower than 0.05 in the univariate analysis, confirmed gender, lower respiratory system infection, and infection with ST147 isolates, but not age or infection with K64 isolates, as risk factors for 30-day mortality. Moreover, the multivariate analysis showed that patients suffering from hematological malignancies or having been treated with inappropriate therapy, both having *p*-values slightly higher than 0.05 in the univariate analysis, exhibited significantly poorer outcomes in the multivariant analysis. The association of the ST147 clone with an increased risk of mortality is a novel finding that deserves further attention. Studies like the one presented here can certainly benefit the management of patients with nosocomial BSI caused by carbapenemase-producing *K. pneumoniae*.

## 1. Introduction

The *Klebsiella* genus comprises various opportunistic pathogenic species, with *K. pneumoniae* being the most clinically significant. This species has the capability to cause a wide range of infections, predominantly affecting hospitalized or immunocompromised patients [1,2]. For a long time, carbapenems have been the last line therapy against severe infections caused by *K. pneumoniae* and other *Enterobacterales*. However, due to the extensive use of these drugs in the past few years, there has been an increase in worldwide dissemination of carbapenem-resistant strains [3]. Resistance to carbapenems in *Enterobacterales* can result from the acquisition of carbapenemase-encoding genes. Currently, OXA-48 is the most prevalent carbapenemase, with widespread distribution in the Middle East, North Africa, Asia, South America, and Europe, particularly in Mediterranean countries [4].

The limited availability of effective drugs against carbapenemase-producing *K. pneumoniae* has made the treatment of nosocomial infections caused by these bacteria increasingly challenging [1,2]. Until recently, when new antibiotics such as cephalosporin combinations with novel beta-lactamase inhibitors were introduced, there were only a handful of drugs, like aminoglycosides, fosfomycin (FF), colistin (CST), or tigecycline (TIG), that could be used to manage these infections. However, these drugs, including carbapenems, have limited effectiveness, encounter resistance issues, and often need to be used in combination therapy, especially when the minimal inhibitory concentration (MIC) to the latter is low [5].

In the case of *Klebsiella* infections, it is crucial to consider not only the presence of resistance determinants but also the virulence factors. *K. pneumoniae* has the capability to cause various severe conditions, including pulmonary and bloodstream infections (BSI). The ability to provoke BSI is associated with the presence of two critical antigens acting as virulence factors: the capsular polysaccharide (K) and the lipopolysaccharide somatic (O) antigens [6]. The K-antigens are also involved in the development of pneumonia, as they confer resistance against phagocytosis by alveolar macrophages and modulate complement C3 deposition [7].

BSI caused by carbapenem-resistant *K. pneumoniae* have high morbidity and mortality rates; therefore, it is important to identify clinical predictors in order to prevent patients at risk from acquiring such infections, and also to recognize patients who would be more predisposed to a poor outcome in order to provide prompt and adequate treatment [8]. Several studies have investigated the clinical risk factors associated with higher mortality in patients with carbapenem-resistant *K. pneumoniae* BSI [9,10,11]. However, reports that have also included microbiological data are still scarce.

The aim of this study was to analyze both the clinical and microbiological factors for 30-day mortality in a cohort of patients with BSI caused by OXA-48-producing *K. pneumoniae*, including the molecular types (ST and serotype), as well as the mechanisms of resistance of the recovered isolates. These isolates were identified, sequenced, and partially characterized in a previous study [12].

## 2. Materials and Methods

### 2.1. Clinical Features of Nosocomial BSI Caused by OXA-48-Producing K. pneumoniae

Over a five-year period (June 2014 to June 2019), 76 BSI were caused by OXA-48-producing *K. pneumoniae* at the Hospital Universitario Central of Asturias (a tertiary Spanish hospital located in northern Spain). A BSI was defined as the presence of viable bacteria in the bloodstream, confirmed by the positivity of one or more blood cultures, but only if this presence led to alterations in clinical, laboratory, and/or hemodynamic parameters and the attending physician treated the patient with antibiotics. Clinical records from the patients were retrospectively reviewed. They included gender, hospital ward (intensive care unit (ICU), medical ward, or surgical ward), Charlson Comorbidity Index, hematological malignancy, and source of infection (catheter-related, intra-abdominal, lower respiratory system, skin and soft tissue, surgical site, urinary system, or unknown). Data regarding the applied therapy were also compiled and categorized into three groups: therapy including carbapenem, combined therapy (defined as the use of two or more different antibiotics simultaneously), and inadequate therapy (defined as treatment that did not include at least one antibiotic to which the bacteria were susceptible according to final antibiogram). The individualized treatment regimens for each patient are detailed in Table S1.

### 2.2. Microbiological Features

All 76 *K. pneumoniae* isolates involved in BSI were partially characterized in a previous study [12]. Briefly, they were screened for carbapenemase production by means of a described algorithm [13], and the *bla*_OXA-48_ gene was detected in all of them. In addition, they were sequenced with short-read Illumina technology and identified experimentally by means of MALDI-TOF/MS (Bruker Daltonics, Billerica, MA, USA) and in silico using the KmerFinder 3.2 tool available at the Center for Genomic Epidemiology (https://www.genomicepidemiology.org/, accessed on 17 January 2020) [14]. For each isolate, the sequence type (ST) was also determined in silico by MLST 2.0 at the CGE.

In the present study, further characterization of the isolates was performed. The Kaptive-Web tool (https://kaptive-web.erc.monash.edu/, accessed on 6 October 2020) was applied to determine the serotype for both the O (somatic) and K (capsular) antigens [15,16]. Results with lower confidence than “Good” were excluded based on the guidelines provided by the developers of the tool. The string test for the detection of hypermucoviscous (HMV) strains related to high virulence was performed for all the isolates as described [17].

In our previous study, the MIC of meropenem (MER), ertapenem (ERT), imipenem (IMP), and cefoxitin (FOX) were already determined, and the genes responsible for the observed resistances (*bla*_OXA-48_ ± BLEE genes) were identified [12]. Additional susceptibility testing was performed for ampicillin (AMP), amoxicillin/clavulanic acid (AMC), piperacillin/tazobactam (P/T), cefotaxime (CTX), cefepime (FEP), ciprofloxacin (CIP), trimethoprim-sulfamethoxazole (SXT), gentamicin (GM), tobramycin (TB), amikacin (AK), and TIG using the Microscan system (Beckman Coulter, Brea, CA). The MIC of CST and FF were determined by broth microdilution according to EUCAST recommendations (www.eucast.org, accessed on 3 March 2020) and by Etest^®^ (bioMérieux, Marcy l’Etoile, France), respectively. ResFinder 4.4.1 allowed for the identification of resistance genes in the genomes sequenced (http://genepi.food.dtu.dk/resfinder, last accessed on 10 November 2023). For CST and FF, the genetic bases of resistance were further investigated through the study of the most common chromosomal mutations described in the literature. FASTA sequences of all the genes of interest were obtained by the use of Clone Manager Professional v9.2 (Sci-Ed Software, Morrisville, NC, USA). Clustal Omega [18] and Jalview [19] were utilized for aligning these sequences with the genome of *K. pneumoniae* ATCC 13883 (accession number JOOW00000000), used as the reference for interpretation purposes. The Protein Variation Effect Analyzer (PROVEAN), a tool capable of predicting the functional impact of amino acid substitutions and indels across various organisms, was employed to foresee deleterious mutations [20].

### 2.3. Phylogenetic Analysis

An SNP-based phylogenetic tree was constructed and visualized using CSI Phylogeny 1.4 (https://cge.food.dtu.dk/services/CSIPhylogeny/, accessed on 26 July 2023), MAFFT version 7 (https://mafft.cbrc.jp/alignment/server/, accessed on 26 July 2023) and Phylo.io (https://phylo.io/, accessed on 26 July 2023). The genome of *K. pneumoniae* ATCC 13883 was used as the reference, and the genome of *K. variicola* Kp_HUCA_Bac_59 was also included for comparison. The latter isolate, recovered from a patient diagnosed with BSI at the HUCA within the period of study, was misidentified as *K. pneumoniae* by MALDI-TOF/MS. However, it was found to belong to *K. variicola* based on a KmerFinder analysis of the sequenced genome. Like the *K. pneumoniae* isolates, it was also positive for *bla*_OXA-48_.

### 2.4. Statistical Analysis

Uni- and multivariate logistic regression analyses were performed to assess the effect of different variables (microbiological features, clinical factors, and therapeutical regimens) in 30-day all-cause mortality using Stata/IC 14.2 2 (StataCorp, College Station, TX, USA) (https://www.stata.com/, accessed on 11 August 2023). The statistical significance was established at *p* < 0.05.

## 3. Results

### 3.1. Phylogenetic Relationships of the Isolates, In Silico Serotyping, and Resistance Properties

**Figure 1:**
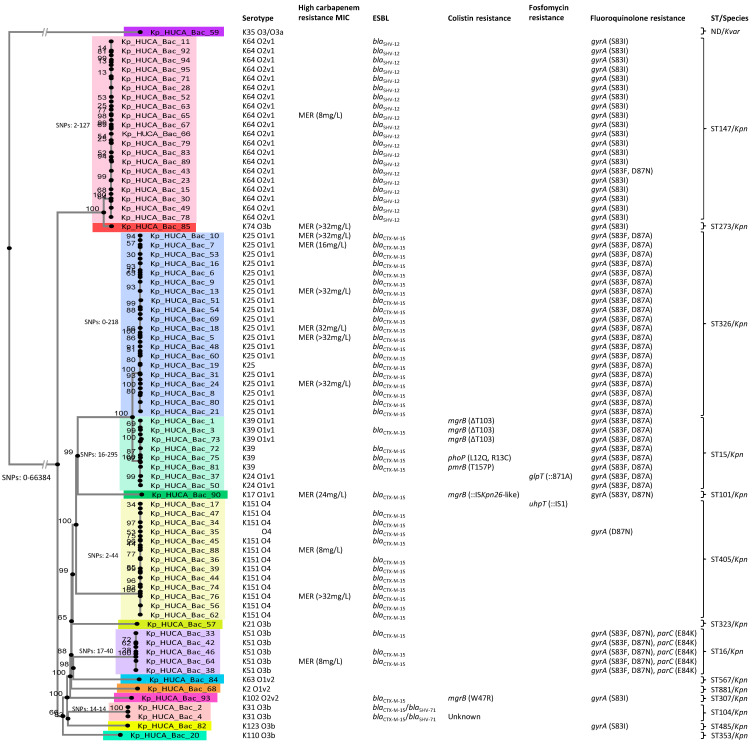
Single nucleotide polymorphism (SNP) tree of 77 *bla*_OXA-48_-carrying *Klebsiella* spp. isolates causing bloodstream infections in a Spanish hospital from 2014 to 2019. Minimum and maximum values of SNP differences of all isolates and within groups of more than one isolate are detailed. Bootstrap values based on 1000 replicates are indicated at the nodes. There are 15 different clusters, one of them corresponding to a *K. variicola* (*Kvar*) isolate and the rest to different sequence types (STs) of *K. pneumoniae* (*Kpn*). Relevant features like serotype (K- and O-antigens), ST, ESBL genes, high MIC of MER, and chromosomal mutations involved in colistin, fosfomycin, and fluoroquinolone resistance are shown. ND, not detected; MIC, minimum inhibitory concentration; MER, meropenem; ESBL, extended-spectrum beta-lactamase; IS, insertion sequence; Δ, deletion. Please note that Kp_HUCA_Bac_59 was first identified as *K. pneumoniae* by MALDI-TOF/MS; therefore, we included the “Kp” label to its identifier, although it was later reclassified as *K. variicola* by KmerFinder.

Among the 76 *K. pneumoniae* isolates, a total of 14 STs were previously identified [12]. ST147 and ST326 were the most common (each accounting for 26.32% of the total isolates), followed by ST405 (17.11%), ST15 (10.53%), ST16 (6.58%), ST104 (2.63%), and ST101, ST273, ST307, ST323, ST353, ST485, ST567, and ST881 (each with a single isolate; 1.32%). The SNP-based phylogenetic tree constructed for the present study distributed the isolates into 14 clusters, which accurately matched the different ST identified for the *K. pneumoniae* isolates (Figure 1). Clusters encompassing ST147 and ST273 isolates appear very close together, consistent with the fact that both ST belong to the same clonal complex, i.e., CG147 [21]. The same situation applies to clusters ST326 and ST15, which belong to clonal complex CG15 [22].

In the phylogenetic tree, a high correlation was also observed between cluster/ST and serotype (Figure 1). For instance, the most prevalent O-antigen, O1v1, shown by 32.47% of the isolates, was shared by all the isolates belonging to ST15, ST101, and ST326. The second most prevalent O-antigen, O2v1, displayed by 25.97% of the isolates was associated with ST147. Other O-antigens detected include O4 (16.88%), O3b (14.29%), O1v2 (2.6%), and O2v2 (1.3%), which were also linked to one or more specific STs (Figure 1). Similarly, each cluster/ST/O-serotype was connected with specific K-antigens, being K25 and K64, shown by ST326 and ST147 isolates, the most prevalent (25.97% each). Additionally, we identified the K2 serotype in a single isolate (Kp_HUCA_Bac_68/ST881). This particular serotype/ST combination has been associated with hypervirulent strains of *K. pneumoniae* [17], previously recognized as HMV [23]. However, the K2 isolate in the present study yielded a negative result in the string test commonly employed to assess the HMV phenotype by observing the formation of a viscous string exceeding 5 mm in length. The O-antigens of Kp_HUCA_Bac_19, Kp_HUCA_Bac_72, Kp_HUCA_Bac_75, and Kp_HUCA_Bac_81, as well as the K-antigen of Kp_HUCA_Bac_35 could not be identified by in silico serotyping. Finally, the *K. variicola* isolate appeared as an outgroup in the phylogenetic tree (Figure 1). There was no ST match for this isolate, which has the O3/O3a and K35 somatic and capsular antigens.

Detailed antimicrobial resistant data, including the resistance phenotypes and responsible genes, are compiled in Table S1. As previously reported [12], all the isolates were resistant to ERT, and 18.18% of them tested non-susceptible to IMP. Approximately 15.58% of the isolates exhibited MIC to MER ≥ 8 mg/L, and they were classified as displaying high carbapenem resistance. Functional alterations in the major OmpK36 porin were identified as the case of the elevated resistance [12]. In addition, all the isolates were resistant to AMP, AMC, and P/T as expected, and most were also resistant to broad spectrum cephalosporins such as CTX (80.52%) and FEP (81.82%), but only 14.29% were resistant to FOX. Resistance to CIP was observed in 88.31% of the isolates, while 77.92% were resistant to SXT. Regarding the aminoglycosides, 55.84% of the isolates were non-susceptible to GM, 53.25% to TB, and 3.90% to AK. Moreover, 24.68% of them exhibited resistance to FF, 7.79% to TIG, and 10.39% to COL. According to these results, all the *K. pneumoniae* isolates were classified as multidrug resistant (MDR) following the criteria defined by Magiorakos et al. [24].

A genome analysis confirmed the presence of the *bla*_OXA-48_ gene in all the isolates. Other oxacillinase-encoding genes were detected, with 57.14% and 28.57% of them carrying *bla*_OXA-1_ and *bla*_OXA-9_, respectively. At least one gene encoding extended-spectrum beta-lactamases (ESBLs) was found in 62 isolates (80.52%). Among them, the *bla*_CTX-M-15_ gene was present in more than half of them (67.74%), while 32.26% and 3.23% carried *bla*_SHV-12_ and *bla*_SHV-71_, respectively. The *bla*_SHV-76_ gene, which encodes a non-ESBL enzyme, was identified in all the ST405 isolates. All the isolates were found to possess both the *oqxA* and *oqxB* genes. Additionally, 44.16% of them were positive for the *aac(6′)-Ib-cr* gene, while 46.75% carried either *qnrB1* or *qnrS1* or both. These results reveal the presence of multiple genes for plasmid-mediated quinolone resistance (PMQR) in the analyzed isolates. Moreover, all of them carried one or more *fosA* allele for FF resistance, although they were not always associated with resistance. Half of the isolates (*n* = 39) carried *fosA* (the first reported *fosA* gene, also known as *fosA1*) and five carried *fosA5* and 32 *fosA6*, while both *fosA6* and *fosA7* were found in a single isolate.

Apart from the detection of acquired resistance genes, a detailed investigation of chromosomal mutations leading to resistance was performed in the present study. Eight out of the total 76 *K. pneumoniae* isolates were resistant to CST according to the broth microdilution method recommended by EUCAST. A bioinformatic analysis revealed that none of them carried a *mcr* gene for plasmid-mediated CST resistance. Five of the eight CST resistant isolates (62.5%) belonged to ST15, while the remaining three were ST101, ST104, or ST307. To investigate the genetic bases of CST resistance, the chromosomal genes most commonly associated with this resistance (*murA*, *pmrA*, *pmrB*, *phoP*, and *phoQ*) were thoroughly analyzed. As shown in Figure 1, a T residue was missing at position 103 of the *mgrB* gene in three ST15 isolates (Kp_HUCA_Bac_1, Kp_HUCA_Bac_3, and Kp_HUCA_Bac_73). In the case of Kp_HUCA_Bac_93 (ST307), a single amino acid change (W47R) was observed in the same gene. Kp_HUCA_Bac_90 (ST101) had an insertion sequence (IS*Kpn26*-like) within the *mgrB* gene, at the 75-nucleotide position. In Kp_HUCA_Bac_81 (ST15), the *pmrB* gene displayed a single amino acid change (T157P). Finally, L12Q and R13C amino acid changes were observed in the PhoP protein of Kp_HUCA_Bac_75 (ST15). No relevant mutations were identified in any of the studied CST-related genes of Kp_HUCA_Bac_4 (ST104); therefore, in this isolate, the genetic background of CST resistance remains unknown.

With regard to fluoroquinolone resistance, apart from identifying multiple PMQR genes, a search of chromosomal mutations in the *gyrA*, *gyrB*, and *parC* genes was conducted [25,26,27]. A total of 75.32% of the isolates harbored at least one deleterious mutation affecting the *gyrA* gene, which led to one or two of the following substitutions in the protein: S83F (44.16%), D87A (36.36%), S83I (28.57%), D87N (10.39%), and S83Y (1.3%). On the other hand, 6.49% of the isolates (all belonging to ST16) exhibited the E84K change in ParC together with a double mutation in *gyrA*. No deleterious mutations were detected on the *gyrB* gene (Figure 1).

A study of mutations in the most relevant chromosomal genes related to FF resistance was also conducted. No mutations were identified in the *murA* gene. However, Kp_HUCA_Bac_37 and Kp_HUCA_Bac_17 carried an adenine insertion at position 871 of *glpT* or an IS*1* inserted at position 1540 of *uhpT*, respectively (Figure 1).

### 3.2. Patient Characteristics and Therapy

The median age of the patients was of 66.7 years, being 53 (68.9%) males. The median Charlson comorbidity score was 5.1 and 30-day all-cause mortality 24/77 (31.2%). More than half of the patients (60.5%) were assigned to the medical ward, 17.1% to the surgical ward, and 22.4% to the ICU. The most common origin of BSI was urinary system infection (28.9%), followed by unknown source (27.6%). Hematological malignancies affected 23.68% of the patients.

In the present series, more than half (56.58%) of the therapies administered included a carbapenem. Combined therapy was used in 64.94% of patients, and 96% of the treatment administered to the patients was deemed adequate.

### 3.3. Risk Factors for 30-Day Mortality

In order to evaluate the possible risk factors associated with 30-day mortality of hospital-acquired BSI caused by OXA-48-producing *K. pneumoniae*, the patient, therapy, and microbiological features were considered (Table 1).

The univariate study unveiled statistically significant correlations between five specific characteristics and 30-day mortality. These factors included age, male gender, lower respiratory system infection, infection by ST147 isolates, and infection by isolates positive for the K64 antigen. Notably, the Charlson Comorbidity Index, hematological malignancies, and inadequate therapy exhibited a trend towards increased 30-day mortality, although these findings did not attain statistical significance. It is worth pointing out that the 20 isolates typed as ST147 matched perfectly with the 20 isolates that carried the K64 antigen. Both the ST147 and K64 isolates were associated with 30-day mortality in the univariate study; however, only ST147 was confirmed as a risk factor for mortality in the multivariate analysis. According to the latter, being a male was also recognized as a risk factor for 30-day mortality. Although there was no specific hospital ward (ICU, medical ward, or surgical ward) linked to a high risk of mortality, patients suffering from hematological malignancies exhibited poorer outcomes (OR 10.5 CI 95% 1.8–61.6, *p* = 0.009). Concerning the source of infection, infections of the lower respiratory system significantly increased 30-day mortality (OR 9.7 CI 95% 1.3–71.5, *p* = 0.025).

Therapeutical features were also contemplated in the multivariate analysis. Regimens including a carbapenem (most strains were susceptible despite being OXA-48 producers) and the use of combined therapy had no impact on mortality. However, inadequate therapy was associated with a 30-day mortality risk (OR 75.8 CI 95% 2.1–2745.6, *p* = 0.018). Regarding the microbiological features, the multivariate analysis revealed that there were no significant associations between any of the K- or O-antigens and 30-day mortality. In contrast, infections caused by the ST147 clone were recognized as a 30-day mortality risk factor (OR 4.5 CI 95% 1.0–20.0, *p* = 0.045).

## 4. Discussion

This is a retrospective single-center clinical study designed to analyze risk factors for 30-day mortality in a cohort of patients with BSI caused by OXA-48-producing *K. pneumoniae* in an endemic setting [28]. In recent years, several studies have investigated mortality predictors in BSI caused by carbapenemase-producing *Enterobacterales* [29,30,31,32]. However, most of them focused on clinical factors, and only a few specifically addressed infections caused by OXA-48-producing *K. pneumoniae* [33,34]. Among the clinical features considered in this study, male sex, hematological malignancy, and a lower respiratory system infection were significantly associated with a higher risk of 30-day mortality. Previous studies have already found that having pneumonia or the use of invasive mechanical ventilation (required by the half of the pneumonia patients in the present study; 6/12) are risk factors associated with high mortality in BSI caused by carbapenemase-producing *Enterobacterales* [34,35]. Notably, there were no significant differences in mortality observed between those on mechanical ventilation (4/6, 66.7%) and those with spontaneous ventilation (3/6, 50%). As reported in the present study, many other authors found that hematological malignancies and/or an immunocompromised status are conditions that increase the risk of mortality [31,36,37,38], although there is variability. An ICU stay was not a factor related to mortality in this series. However, it is important to highlight that in the context of hematological patients with advanced disease, there is a restraint on therapeutic interventions. In this specific patient cohort, the overall mortality rate was elevated, with admission to the ICU being infrequent. Even if these patients were admitted to the ICU, the anticipated outcome would likely have been comparable, potentially contributing to an increase in mortality statistics among the ICU patients.

As expected, and in agreement with previous findings [11,39,40], inadequate regimens were associated with higher risk of 30-day mortality. It is worth noting that the episodes analyzed in our series occurred at a time when most European hospitals did not have access to new beta-lactams with anti-carbapenemase activity. Additionally, the guidelines at the time recommended the use of combined therapy, especially in patients at risk or when carbapenem MICs were high [5,9]. In the present study, most isolates were susceptible to MER, displaying low MIC values, which is common in OXA-48-producing *Enterobacterales*. This fact could justify the relatively low use of combined therapy in our series (applied to 64.94% of the patients). Papadimitriou-Olivgeris et al. [40] identified that in critically ill patients suffering from BSI caused by KPC-producing *K. pneumoniae*, combined therapy was a predictor of a good prognosis. In addition, Chen et al. [39] reported that combined therapy with a high dose of carbapenem was related to better outcomes, while MICs to MER of more than 8 mg/L were associated with higher mortality. However, in the present study, neither the use of combined therapy nor a high MIC of MER was related to an increase in patient mortality. The variability in different studies with respect to the benefit of combined therapy could be due to factors such as the type of carbapenemase, the susceptibility to the different drugs combined, and/or the patient’s comorbidity/severity, as the INCREMENT study reported [9]. Regarding the antimicrobials employed in the combined therapy within this series, a statistical analysis was not performed due to the substantial variability observed in prescribed regimens, encompassing diverse antibiotics and dosages (see Table S1). The majority of the combined therapies comprised two or three antibiotics, including carbapenems, aminoglycosides, colistin, and/or tigecycline. As previously stated, the strength of the present study relies on the inclusion in the analysis not only of clinical features, but also of important microbiological factors, such as the ST and serotype of the isolates, which could influence the patient’s outcome, and which were available thanks to the sequencing of their whole genomes. Few studies have reported that certain clones are predictors of mortality in infections caused by carbapenemase-producing *K. pneumoniae.* For instance, in one of the few previous works that included microbiological factors as predictors of mortality, infection by ST11 carbapenem-resistant *K. pneumoniae* was linked to high risk 30-day mortality [41]. In our series, 14 distinct STs were identified among the *K. pneumoniae* isolates. This high diversity is remarkable, considering that they all came from the same hospital, but confirm previous observations obtained for 2015 [28]. The MLST results correlated very well with the SNP-based phylogenetic tree built for the present study (Figure 1). High-risk clones, such as ST147, ST15, ST307, and ST101 [42,43], were identified among our isolates. Interestingly, ST147 was associated with a higher risk of 30-day mortality in our series, a finding that gains significance given the extensive distribution of patients infected by this clone across up to 10 different hospital wards. Although ST147 is a well-known high-risk virulent epidemic clone characterized by resistance to carbapenems and by the carriage of the *bla*_SHV-12_ gene [42], its association with a high mortality risk in BSI is a novel finding. Additional studies that include a larger number of patients would be required to further corroborate this observation.

The analysis of the capsular types of the isolates also revealed great diversity, although no statistical significance was found regarding their impact on mortality in the multivariant analysis. In our study, we identified a single isolate with the K2 serotype that belonged to ST881, a clone previously recognized as HMV and associated with hypervirulent strains of *K. pneumoniae* [23]. This particular serotype is known to exhibit increased resistance to phagocytosis due to the structure of the capsule, which consists of glycoepitopes that are not recognized by components of the innate immune system [44]. However, the HMV phenotype of the K2 isolate in the present study could not be demonstrated, and the patient who suffered BSI caused by this isolate survived.

In addition to information regarding the STs and serotypes, whole-genome sequencing of the isolates provided valuable insight on the molecular bases of their resistance mechanisms to different antibiotics. In a previous study, the genetic bases of carbapenem resistance were determined, showing the presence of *bla*_OXA-48_ in all the BSI isolates and identifying multiple alterations in OmpK36 as being responsible for high MER resistance in some of them [12]. Moreover, ESBL genes were detected in 80.52% of the isolates, further complicating the choice of an appropriate therapy. Within this scenario, the identification of CST-resistant isolates recovered from patients with BSI is particularly worrisome. Most of the CST-resistant isolates belonged to the high-risk clones ST15 and ST101, and one of them also displayed high carbapenem resistance, challenging the treatment of the patient from which it was recovered. Although *mcr* genes were not detected, different alterations in three regulatory genes, *mgrB*, *phoP*, and *pmrB*, known to be involved in CST resistance, were identified [45]. Most of these alterations have been previously associated with CST resistance in the literature [46,47]. However, we found two amino acid changes in the *phoP* gene of Kp_HUCA_Bac_75 (L12Q and R13C) that were not previously reported together as conferring CST resistance but were predicted to be deleterious by the PROVEAN v1.1.5 software [48]. Nonetheless, the L12Q substitution was recently identified in a CST-resistant *K. pneumoniae* isolate in France [49].

The high prevalence of fluoroquinolone-resistant strains (88.31%), associated with a very high number of PMQR genes and chromosomal mutations affecting the *gyrA* and *parC* genes, is also worrisome. Interestingly, all the ST147 isolates carried the widely reported S83I substitution in GyrA [42], and one of them contained the additional D87N amino acid change. Several isolates were resistant to FF (24.68%), one of the drugs used in combined therapy against infections caused by carbapenemase-producing *Enterobacterales* [5]. Various mechanisms have evolved in bacteria to impede its effectiveness, including the presence of resistance genes such as *fosA*, which is intrinsically present in *K. pneumoniae,* although its expression does not always translate into FF in vitro resistance [50]. Additionally, mutations in key factors involved in peptidoglycan biosynthesis, like *murA*, or in transport genes, like *uhpT* or *glpT*, can also impact FF efficacy. The products of these genes facilitate the active entry of FF into the cell [51,52]. The susceptibility of Kp_HUCA_Bac_37 to FF, despite the presence of a frameshift mutation in *glpT*, may be attributed to the potential masking of *glpT* mutations by the proper expression of *uhpT* [53]. In this study, 100% of the isolates harbored a plasmid-borne *fosA* gene or one of its alleles; nonetheless, only 24.68% of the strains were resistant to FF.

While the present study yields compelling insights, it is crucial to acknowledge its limitations, including a single-center, retrospective design and a small sample size. Additionally, the definition of inadequate treatment was oversimplified, focusing solely on coverage by at least one antibiotic appropriate for the identified causal agent. Nonetheless, the extensive variability in therapeutic regimens, encompassing diverse antibiotics and dosages, hindered the possibility of conducting a statistical analysis to assess the specific impact of each treatment on mortality.

## 5. Conclusions

The present study provides and integrates relevant microbiological and clinical information, which complements the data currently available in the scientific literature on predictors of mortality associated with BSI caused by carbapenemase-producing *K. pneumoniae.* Of particular interest and novelty is the association of the ST147 clone with a high mortality risk. Our findings also emphasize the complex nature of antibiotic resistance and the importance of an appropriate empirical treatment for a positive patient outcome. The retrospective, observational, and single-center design of the study can be a limitation. Thus, further research, including a higher number of patients from different hospitals and the incorporation of additional variables, will be required, as it will certainly benefit the management of patients with nosocomial BSI caused by carbapenemase-producing *K. pneumoniae*.

## Figures and Tables

**Table 1:** Risk factors for 30-day mortality of bloodstream infections caused by OXA-48-producing Klebsiella pneumoniae.

	Dead (*n* = 24)	Survival (*n* = 52)	Univariate Analysis		Multivariate Analysis ^1^	
			OR (CI 95%)	*p*	OR (CI 95%)	*p*
Clinical features						
Age ^2^			1.5 (1.0–2.2)	**0.042**	1.5 (0.8–2.8)	0.197
Gender *n* (%)						
Female	2 (8.3)	21 (40.4)	0.1 (0.0–0.6)	**0.009**	0.1 (0.0–0.7)	**0.026**
Male	22 (91.7)	31 (56.4)	1		1	
Hospital ward *n* (%)						
ICU	8 (33.3)	9 (17.3)	2 (0.4–9.1)	0.370	-	
Medical	12 (50.0)	34 (65.4)	0.8 (0.2–3.1)	0.738	-	
Surgical	4 (16.7)	9 (17.3)	1		-	
Charlson ^3^ *n* (%)			1.2 (1.0–1.5)	**0.063**	1.2 (0.9–1.7)	0.238
Hematological malignancy	9 (37.5)	9 (17.3)	2.9 (1.0–8.6)	**0.059**	10.5 (1.8–61.6)	**0.009**
Source of infection ^4^ *n* (%)						
Catheter-related infection	2 (8.3)	2 (3.9)	4.5 (0.5–42.2)	0.188	-	
Intra-abdominal infection	1 (4.2)	10 (19.2)	0.5 (0.0–4.6)	0.501	-	
Lower respiratory system infection	7 (29.2)	5 (9.6)	6.3 (1.3–30.5)	**0.022**	9.7 (1.3–71.5)	**0.025**
Skin and soft tissue infection	1 (4.2)	0	1		-	
Surgical site infection	1 (4.2)	4 (7.7)	1.1 (0.1–13)	0.925	-	
Unknown	8 (33.3)	13 (25.0)	2.8 (0.7–11.2)	0.153	-	
Urinary system infection	4 (16.7)	18 (34.7)	1		-	
Therapy features						
Therapy included carbapenem ^5^	14 (58.3)	29 (55.8)	1.1 (0.4–3.0)	0.834	-	
Combined therapy ^5^	16 (66.7)	34 (65.4)	1.1 (0.4–3.0)	0.913	-	
Inadequate therapy ^6^	3 (12.5)	1 (1.9)	7.3 (0.7–74.1)	**0.093**	75.8 (2.1–2745.6)	**0.018**
Microbiological features						
High carbapenem resistance	2 (8.3)	10 (19.2)	0.4 (0.1–1.9)	0.239		
Clone						
ST147	10 (41.7)	10 (19.2)	6.7 (1.5–29.8)	**0.013**	4.5 (1.0–20.0)	**0.045**
ST326	7 (29.2)	13 (25)	3.6 (0.8–16.4)	0.100	-	
ST405	4 (16.7)	9 (17.3)	3 (0.5–16.1)	0.208	-	
Other ^7^	3 (12.5)	21 (38.5)	1		-	
K-antigen						
K151	4 (16.7)	7 (13.5)	3.8 (0.7–21.4)	0.129	-	
K25	7 (29.2)	14 (26.9)	3.3 (0.7–15.2)	0.119	-	
K64	10 (41.7)	10 (19.2)	6.7 (1.5–29.8)	**0.013**	1	
Other ^8^	3 (12.5)	21 (40.4)	1		-	
O-antigen						
O1	6 (27.3)	22 (44.0)	0.5 (0.1–2.4)	0.429	-	
O2	10 (45.5)	11 (22.0)	1.8 (0.4–7.9)	0.427	-	
O3	2 (9.1)	9 (18.0)	0.4 (0.1–3.1)	0.414	-	
O4	4 (16.0)	8 (18.2)	1		-	

ICU, intensive care unit; OR, odds ratio; CI, confidence interval. ^1^ Multivariate analysis was applied to variables that showed *p*-values lower or slightly higher than 0.05. These values are highlighted in bold in the univariate column, and the same applies to the significative *p*-values in the multivariate column. ^2^ Patients were grouped by age considering decades. ^3^ Charlson score was analyzed as a continuous variable. ^4^ CDC definitions for specific types of infections were used as standards. ^5^ New antibiotics with activity against carbapenemases (ceftazidime-avibactam, meropenem-vaborbactam, etc.) were not available at our hospital during the time of the study; therefore, the use of combined therapy, frequently including a carbapenem, was the clinical standard according to the guidelines. A treatment was considered as combined when it included at least two antibiotics to which the bacterial isolate was susceptible according to antibiogram. ^6^ Therapy was considered incorrect when it did not include at least one antibiotic to which the isolate was susceptible according to antibiogram. ^7^ Other STs included ST15, ST16, ST101, ST104, ST273, ST307, ST323, ST353, ST485, ST567, and ST881. ^8^ Other K-antigens included K2, K17, K21, K31, K35, K39, K63, K74, K102, K110, and K123.

## Data Availability

The genomes of all the isolates studied in this work have been deposited in GenBank and are available within the BioProject PRJNA718833. The accession numbers for the *K. pneumoniae* isolates are compiled in Supplementary Table S1 of a previous study [12]. The accession number for the *K. variicola* strain (Kp_HUCA_Bac_59) is JAGKYQ000000000.

## Supplementary Materials

The following supporting information can be downloaded at: https://www.mdpi.com/article/10.3390/pathogens13010011/s1, Table S1: Molecular features of *bla*_OXA-48_-carrying *Klebsiella* spp. isolates recovered from blood cultures in a Spanish hospital from 2014 to 2019.
