# Supplementary material for: Clinical and Microbiological Risk Factors for 30-Day Mortality of Bloodstream Infections Caused by OXA-48-Producing Klebsiella pneumoniae
Source: Pathogens. 2023 Dec 21;13(1):11. doi: 10.3390/pathogens13010011 (PMC10819929; doi:10.3390/pathogens13010011)
Supplement: Supplementary file 1 [file pathogens-13-00011-s001.zip › pathogens-2760889-supplementary.pdf]

pathogens

MDPI

Article

Clinical and Microbiological Risk Factors for 30-Day Mortality of Bloodstream Infections Caused by OXA-48-Producing *Klebsiella pneumoniae*

Pilar Lumbreras-Iglesias [1,2,3], Edurne Rodrigo-Arrazola [2], Lucía López-Amor [4], Jonathan Fernández-Suárez [1,2], María Rosario Rodicio [1,5,*,†] and Javier Fernández [1,2,5,6,7,*,†]

[1] Traslational Microbiology Group, Health Research Institute of the Principality of Asturias (ISPA), 33011 Oviedo, Spain; uo231178@uniovi.es (P.L.-I.); jhonatan.fernandez@sespa.es (J.F.-S.)
[2] Department of Clinical Microbiology, Central University Hospital of Asturias (HUCA), 33011 Oviedo, Spain; edurnearrazola@gmail.com
[3] Hematological malignancies Group, Health Research Institute of the Principality of Asturias (ISPA), 33011 Oviedo, Spain;
[4] Department of Intensive Care Medicine, San Agustín University Hospital (HUSA), 33401 Avilés, Spain; lucia.lopez@sespa.es
[5] Department of Functional Biology, Microbiology Area, University of Oviedo, 33006 Oviedo, Spain
[6] Research & Innovation, Artificial Intelligence and Statistical Department, Pragmatech AI Solutions, 33001 Oviedo, Spain
[7] Biomedical Research Networking Center - Respiratory diseases, 28029 Madrid, Spain
* Correspondence: rrodicio@uniovi.es (M.R.R.); fernandezdjavier@uniovi.es (J.F.)
† These authors contributed equally to this work.

Table S1: Administering therapy to patients and molecular features of *bla*$_{OXA-48}$-carrying *Klebsiella* spp. isolates recovered from blood cultures in a Spanish hospital from 2014 to 2019.

Isolate	Resistance genes	Antimicrobial resistance	Antimicrobial therapy
Kp_HUCA_Bac_1	*bla*$_{OXA-1}$, *bla*$_{OXA-48}$, *bla*$_{SHV-28}$, *aac(6')-Ib-cr*, *oqxA*, *oqxB*, *fosA6*, *aadA2*, *aph(3')-Ia*, *sul1*, *mph(A)*, *dfrA12*, *catB3*	AMP, AMC, P/T, ERT, CIP, SXT, TB, CST	Meropenem + Tigecycline
Kp_HUCA_Bac_2	*bla*$_{CTX-M-15}$, *bla*$_{OXA-1}$, *bla*$_{OXA-48}$, *bla*$_{OXA-9}$, *bla*$_{SHV-71}$, *bla*$_{TEM-1A}$, *oqxA*, *oqxB*, *fosA*, *aac(3)-IIa*, *aac(6')-Ib*, *aac(6')-Ib-cr*, *aac(6')-Ib3*, *aadA1*, *drfA14*, *catB3*, *tet(D)*	AMP, AMC, P/T, CTX, FEP, ERT, SXT, GM, TB	Meropenem + Amikacin
Kp_HUCA_Bac_3	*bla*$_{CTX-M-15}$, *bla*$_{OXA-1}$, *bla*$_{OXA-48}$, *bla*$_{SHV-28}$, *bla*$_{TEM-1B}$, *aac(6')-Ib-cr*, *oqxA*, *oqxB*, *fosA6*, *aadA2*, *aph(3')-Ia*, *sul1*, *mph(A)*, *dfrA12*, *catB3*	AMP, AMC, P/T, CTX, FEP, ERT, CIP, SXT, TB, CST	Imipenem + Gentamicin + Septrin
Kp_HUCA_Bac_4	*bla*$_{CTX-M-15}$, *bla*$_{OXA-1}$, *bla*$_{OXA-48}$, *bla*$_{OXA-9}$, *bla*$_{SHV-71}$, *bla*$_{TEM-1A}$, *oqxA*, *oqxB*, *fosA*, *aac(3)-IIa*, *aac(6')-Ib3*, *aadA1*, *dfrA14*, *catB3*, *tet(D)*	AMP, AMC, P/T, CTX, FEP, ERT, TB, CST	Meropenem
Kp_HUCA_Bac_5	*bla*$_{CTX-M-15}$, *bla*$_{OXA-1}$, *bla*$_{OXA-48}$, *bla*$_{OXA-9}$, *bla*$_{SHV-28}$, *bla*$_{TEM-1A}$, *oqxA*, *oqxB*, *fosA6*, *aac(3)-IIa*, *aac(6')-Ib3*, *aadA1*, *dfrA14*, *catB3*	AMP, AMC, P/T, FOX, CTX, FEP, ERT, IMP, MER, CIP, SXT, GM, TB, AK, FF, TIG	Colistin
Kp_HUCA_Bac_6	*bla*$_{CTX-M-15}$, *bla*$_{OXA-1}$, *bla*$_{OXA-48}$, *bla*$_{OXA-9}$, *bla*$_{SHV-28}$,	AMP, AMC, P/T, CTX,	Meropenem +

	*bla*TEM-1A, *oqxA, oqxB, fosA6, aac(6′)-Ib3, aadA1, dfrA14, catB3*	FEP, ERT, CIP, SXT, TB, FF	Piperacillin/Tazobactam + Amikacin
Kp_HUCA_Bac_7	*bla*CTX-M-15, *bla*OXA-1, *bla*OXA-48, *bla*OXA-9, *bla*SHV-28, *bla*TEM-1A, *aac(6′)-Ib-cr, oqxA, oqxB, fosA6, aac(3)-IIa, aac(6′)-Ib, aadA1, dfrA14, catB3, tet(D)*	AMP, AMC, P/T, FOX, FEP, ERT, IMP, MER, CIP, SXT, GM, TB, FF	Meropenem + Amikacin + Tigecycline
Kp_HUCA_Bac_8	*bla*CTX-M-15, *bla*OXA-1, *bla*OXA-48, *bla*OXA-9, *bla*SHV-1, *bla*TEM1A, *aac(6′)-Ib-cr, oqxA, oqxB, fosA6, aac(6′)-Ib, aac(6′)-Ib3, aadA1, catB3, tet(D)*	AMP, AMC, P/T, CTX, FEP, ERT, CIP, TB	Amikacin + Imipenem + Colistin
Kp_HUCA_Bac_9	*bla*CTX-M-15, *bla*OXA-1, *bla*OXA-48, *bla*OXA-9, *bla*SHV-28, *bla*TEM-1A, *oqxA, oqxB, fosA6, aac(6′)-Ib3, aadA1, dfrA14, catB3*	AMP, AMC, P/T, CTX, FEP, ERT, CIP, SXT, TB, FF	Meropenem + Colistin
Kp_HUCA_Bac_10	*bla*CTX-M-15, *bla*OXA-1, *bla*OXA-48, *bla*OXA-9, *bla*SHV-28, *bla*TEM-1A, *aac(6′)-Ib-cr, oqxA, oqxB, fosA6, aac(3)-IIa, aac(6′)-Ib, aadA1, dfrA14, catB3, tet(D)*	AMP, AMC, P/T, FOX, CTX, FEP, ERT, IMP, MER, CIP, SXT, TB, FF	Meropenem
Kp_HUCA_Bac_11	*bla*OXA-48, *bla*SHV-12, *bla*TEM-1B, *oqxA, oqxB, qnrB1, fosA, aac(3)-IId, aph(3′′)-Ib, aph(6)-Id, sul2, dfrA14, catA2, tet(A), tet(D)*	AMP, AMC, P/T, CTX, FEP, ERT, CIP, SXT, GM	Colistin + Amikacin
Kp_HUCA_Bac_13	*bla*CTX-M-15, *bla*OXA-1, *bla*OXA-48, *bla*OXA-9, *bla*SHV-28, *bla*TEM-1A, *oqxA, oqxB, fosA6, aac(6′)-Ib3, aadA1, dfrA14, catB3, tet(D)*	AMP, AMC, P/T, FOX, CTX, FEP, ERT, IMP, MER, CIP, SXT, TB, AK, FF	Colistin
Kp_HUCA_Bac_15	*bla*OXA-48, *bla*SHV-12, *bla*TEM-1B, *oqxA, oqxB, qnrB1, fosA, aac(3)-IId, aph(3′′)-Ib, aph(6)-Id, sul2, dfrA14, catA2, tet(A), tet(D)*	AMP, AMC, P/T, CTX, FEP, ERT, CIP, SXT, GM	Colistin
Kp_HUCA_Bac_16	*bla*CTX-M-15, *bla*OXA-1, *bla*OXA-48, *bla*OXA-9, *bla*SHV-28, *bla*TEM-1A, *aac(6′)-Ib-cr, oqxA, oqxB, fosA6, aac(3)-IIa, aac(3′)-Ib3, aadA1, dfrA14, catB3, tet(D)*	AMP, AMC, P/T, CTX, FEP, ERT, CIP, SXT, GM, TB, FF, TIG	Imipenem
Kp_HUCA_Bac_17	*bla*OXA-48, *bla*SHV-76, *oqxA, oqxB, qnrB1, fosA, dfrA14*	AMP, AMC, P/T, ERT, CIP, SXT, FF	Piperacillin/Tazobactam
Kp_HUCA_Bac_18	*bla*CTX-M-15, *bla*OXA-1, *bla*OXA-48, *bla*OXA-9, *bla*SHV-28, *bla*TEM-1A, *oqxA, oqxB, fosA6, aac(6′)-Ib3, aadA1, dfrA14, catB3, tet(D)*	AMP, AMC, P/T, FOX, CTX, FEP, ERT, IMP, MER, CIP, SXT, TB, FF	Colistin
Kp_HUCA_Bac_19	*bla*CTX-M-15, *bla*OXA-1, *bla*OXA-48, *bla*OXA-9, *bla*SHV-28, *bla*TEM-1A, *oqxA, oqxB, fosA6, aac(3)-IIa, aac(6′)-Ib3, aadA1, dfrA14, catB3, tet(D)*	AMP, AMC, P/T, CTX, FEP, ERT, CIP, SXT, GM, TB	Amikacin
Kp_HUCA_Bac_20	*bla*OXA-48, *bla*SHV-1, *oqxA, oqxB, fosA6, tet(D)*	AMP, AMC, P/T, ERT	Ciprofloxacin
Kp_HUCA_Bac_21	*bla*CTX-M-15, *bla*OXA-1, *bla*OXA-48, *bla*OXA-9, *bla*SHV-28, *bla*TEM-1A, *aac(6′)-Ib-cr, oqxA, oqxB, fosA6, aac(6′)-Ib3, aadA1, dfrA14, catB3, tet(D)*	AMP, AMC, P/T, CTX, FEP, ERT, CIP, TB	Imipenem + Colistin

Kp_HUCA_Bac_23	*bla*$_{OXA-48}$, *bla*$_{SHV-12}$, *bla*$_{TEM-1B}$, *oqxA, oqxB, qnrB1, fosA, aac(3)-IId, aph(3'')-Ib, aph(6)-Id, sul2, dfrA14, catA2, tet(A), tet(D)*	AMP, AMC, P/T, CTX, FEP, ERT, CIP, SXT, GM	Amikacin + Colistin
Kp_HUCA_Bac_24	*bla*$_{CTX-M-15}$, *bla*$_{OXA-1}$, *bla*$_{OXA-48}$, *bla*$_{OXA-9}$, *bla*$_{SHV-1}$, *bla*$_{TEM-1A}$, *aac(6')-Ib-cr, oqxA, oqxB, fosA6, aac(6')-Ib, aac(6')-Ib3, aadA1, dfrA14, catB3, tet(D)*	AMP, AMC, P/T, FOX, CTX, FEP, ERT, IMP, MER, CIP, SXT, TB, FF	Gentamicin + Colistin
Kp_HUCA_Bac_28	*bla*$_{OXA-48}$, *bla*$_{SHV-12}$, *bla*$_{TEM-1B}$, *oqxA, oqxB, qnrB1, fosA, aph(3'')-Ib, aph(6)-Id, sul2, dfrA14, catA2, tet(D)*	AMP, AMC, P/T, CTX, FEP, ERT, CIP, SXT	Meropenem + Colistin
Kp_HUCA_Bac_30	*bla*$_{OXA-48}$, *bla*$_{SHV-12}$, *bla*$_{TEM-1B}$, *oqxA, oqxB, qnrB1, fosA, aph(3'')-Ib, aph(6)-Id, sul2, dfrA14, catA2, tet(A), tet(D)*	AMP, AMC, P/T, CTX, FEP, ERT, CIP, SXT	Amikacin + Colistin
Kp_HUCA_Bac_31	*bla*$_{CTX-M-15}$, *bla*$_{OXA-1}$, *bla*$_{OXA-48}$, *bla*$_{OXA-9}$, *bla*$_{SHV-28}$, *bla*$_{TEM-1A}$, *aac(6')-Ib-cr, oqxA, oqxB, fosA6, aac(3)-IIa, aac(6')-Ib, aac(6')-Ib3, aadA1, dfrA14, catB3, tet(D)*	AMP, AMC, P/T, CTX, FEP, ERT, CIP, SXT, GM, TB, TIG	Imipenem + Amikacin
Kp_HUCA_Bac_33	*bla*$_{CTX-M-15}$, *bla*$_{OXA-1}$, *bla*$_{OXA-48}$, *bla*$_{SHV-1}$, *aac(6')-Ib-cr, oqxA, oqxB, fosA5, aadA2, aph(3')-Ia, sul1, mph(A), dfrA12, catB3*	AMP, AMC, P/T, CTX, FEP, ERT, CIP, SXT	Meropenem + Gentamicin
Kp_HUCA_Bac_34	*bla*$_{CTX-M-15}$, *bla*$_{OXA-1}$, *bla*$_{OXA-48}$, *bla*$_{SHV-76}$, *bla*$_{TEM-1B}$, *aac(6')-Ib-cr, oqxA, oqxB, qnrB1, fosA, aac(3)-IIa, aph(3'')-Ib, aph(6)-Id, sul2, dfrA14, catB3, tet(A)*	AMP, AMC, P/T, CTX, FEP, ERT, CIP, SXT, GM, TB	Colistin + Amikacin
Kp_HUCA_Bac_35	*bla*$_{CTX-M-15}$, *bla*$_{OXA-1}$, *bla*$_{OXA-48}$, *bla*$_{SHV-76}$, *bla*$_{TEM-1B}$, *aac(6')-Ib-cr, oqxA, oqxB, qnrB1, fosA, aac(3)-IIa, aph(3'')-Ib, aph(6)-Id, sul2, dfrA14, catB3, tet(A)*	AMP, AMC, P/T, CTX, FEP, ERT, CIP, SXT, GM, TB	Meropenem + Colistin
Kp_HUCA_Bac_36	*bla*$_{CTX-M-15}$, *bla*$_{OXA-1}$, *bla*$_{OXA-48}$, *bla*$_{SHV-76}$, *bla*$_{TEM-1B}$, *aac(6')-Ib-cr, oqxA, oqxB, qnrB1, fosA, aac(3)-IIa, aph(3'')-Ib, aph(6)-Id, sul2, dfrA14, catB3, tet(A)*	AMP, AMC, P/T, CTX, FEP, ERT, CIP, SXT, GM, TB	Colistin + Amikacin
Kp_HUCA_Bac_37	*bla*$_{OXA-48}$, *bla*$_{SHV-28}$, *oqxA, oqxB, fosA6*	AMP, AMC, P/T, ERT, CIP	Cefotaxime
Kp_HUCA_Bac_38	*bla*$_{OXA-48}$, *bla*$_{SHV-1}$, *oqxA, oqxB, fosA5*	AMP, AMC, P/T, ERT, CIP	Meropenem
Kp_HUCA_Bac_39	*bla*$_{CTX-M-15}$, *bla*$_{OXA-1}$, *bla*$_{OXA-48}$, *bla*$_{SHV-76}$, *bla*$_{TEM-1B}$, *aac(6')-Ib-cr, oqxA, oqxB, qnrB1, fosA, aac(3)-IIa, aph(3'')-Ib, aph(6)-Id, sul2, dfrA14, catB3, tet(A)*	AMP, AMC, P/T, CTX, FEP, ERT, CIP, SXT, GM, TB	Colistin + Amikacin
Kp_HUCA_Bac_42	*bla*$_{OXA-48}$, *bla*$_{SHV-1}$, *oqxA, oqxB, fosA5*	AMP, AMC, P/T, ERT, CIP	Amikacin
Kp_HUCA_Bac_43	*bla*$_{OXA-48}$, *bla*$_{SHV-12}$, *bla*$_{TEM-1B}$, *oqxA, oqxB, qnrB1, fosA, aac(3)-IId, aph(3'')-Ib, aph(6)-Id, sul2, dfrA14, catA2, tet(A), tet(D)*	AMP, AMC, P/T, CTX, FEP, ERT, CIP, SXT, GM	Imipenem + Colistin

Kp_HUCA_Bac_44	*bla*$_{CTX-M-15}$, *bla*$_{OXA-1}$, *bla*$_{OXA-48}$, *bla*$_{SHV-76}$, *bla*$_{TEM-1B}$, *aac(6′)-Ib-cr, oqxA, oqxB, qnrB1, fosA, aac(3)-IIa, aph(3′′)-Ib, aph(6)-Id, sul2, dfrA14, catB3, tet(A)*	AMP, AMC, P/T, CTX, FEP, ERT, CIP, SXT, GM, TB	Meropenem
Kp_HUCA_Bac_45	*bla*$_{CTX-M-15}$, *bla*$_{OXA-1}$, *bla*$_{OXA-48}$, *bla*$_{SHV-76}$, *bla*$_{TEM-1B}$, *aac(6′)-Ib-cr, oqxA, oqxB, fosA, aac(3)-IIa, aph(3′′)-Ib, aph(6)-Id, sul2, dfrA14, catB3*	AMP, AMC, P/T, CTX, FEP, ERT, SXT, GM, TB	Meropenem + Amikacin
Kp_HUCA_Bac_46	*bla*$_{CTX-M-15}$, *bla*$_{OXA-1}$, *bla*$_{OXA-48}$, *bla*$_{SHV-1}$, *aac(6′)-Ib-cr, oqxA, oqxB, fosA5, aadA2, aph(3′)-Ia, sul1, mph(A), dfrA12, catB3*	AMP, AMC, P/T, CTX, FEP, ERT, CIP, SXT	Amikacin + Colistin
Kp_HUCA_Bac_47	*bla*$_{CTX-M-15}$, *bla*$_{OXA-1}$, *bla*$_{OXA-48}$, *bla*$_{SHV-76}$, *aac(6′)-Ib-cr, oqxA, oqxB, fosA, aac(3)-IIa, dfrA14, catB3*	AMP, AMC, P/T, CTX, FEP, ERT, CIP, SXT, GM, TB	Meropenem + Colistin
Kp_HUCA_Bac_48	*bla*$_{CTX-M-15}$, *bla*$_{OXA-1}$, *bla*$_{OXA-48}$, *bla*$_{OXA-9}$, *bla*$_{SHV-28}$, *bla*$_{TEM-1A}$, *aac(6′)-Ib-cr, oqxA, oqxB, fosA6, aac(3)-IIa, aac(6′)-Ib, aadA1, dfrA14, catB3, tet(D)*	AMP, AMC, P/T, CTX, FEP, ERT, CIP, SXT, GM, TB	Meropenem + Amikacin
Kp_HUCA_Bac_49	*bla*$_{OXA-48}$, *bla*$_{SHV-12}$, *bla*$_{TEM-1B}$, *oqxA, oqxB, qnrB1, fosA, aac(3)-IId, aph(3′′)-Ib, aph(6)-Id, sul2, dfrA14, catA2, tet(A), tet(D)*	AMP, AMC, P/T, CTX, FEP, ERT, CIP, SXT, GM, TB, TIG	Meropenem + Amikacin
Kp_HUCA_Bac_50	*bla*$_{OXA-48}$, *bla*$_{SHV-28}$, *oqxA, oqxB, fosA6*	AMP, AMC, P/T, ERT, CIP, FF	Gentamicin
Kp_HUCA_Bac_51	*bla*$_{CTX-M-15}$, *bla*$_{OXA-1}$, *bla*$_{OXA-48}$, *bla*$_{OXA-9}$, *bla*$_{SHV-28}$, *bla*$_{TEM-1A}$, *aac(6′)-Ib-cr, oqxA, oqxB, fosA6, aac(3)-IIa, aac(6′)-Ib, aadA1, dfrA14, catB3*	AMP, AMC, P/T, CTX, FEP, ERT, CIP, SXT, GM, TB, AK	Colistin
Kp_HUCA_Bac_52	*bla*$_{OXA-48}$, *bla*$_{SHV-12}$, *bla*$_{TEM-1B}$, *oqxA, oqxB, qnrB1, fosA, aac(3)-IId, aph(3′′)-Ib, aph(6)-Id, sul2, dfrA14, catA2, tet(A), tet(D)*	AMP, AMC, P/T, CTX, FEP, ERT, CIP, SXT, GM	Amikacin
Kp_HUCA_Bac_53	*bla*$_{CTX-M-15}$, *bla*$_{OXA-1}$, *bla*$_{OXA-48,}$ *bla*$_{OXA-9}$, *bla*$_{SHV-28,}$ *bla*$_{TEM-1A}$, *oqxA, oqxB, fosA6, aac(3)-IIa, aac(6′)-Ib3, aadA1, dfrA14, catB3, tet(D)*	AMP, AMC, P/T, CTX, FEP, ERT, CIP, SXT, GM, TB	Meropenem + Colistin
Kp_HUCA_Bac_54	*bla*$_{CTX-M-15}$, *bla*$_{OXA-1}$, *bla*$_{OXA-48}$, *bla*$_{OXA-9}$, *bla*$_{SHV-28}$, *bla*$_{TEM-1A}$, *oqxA, oqxB, fosA6, aac(3)-IIa, aac(6′)-Ib3, aadA1, dfrA14, catB3*	AMP, AMC, P/T, FOX, CTX, FEP, ERT, CIP, SXT, GM, TB	Meropenem + Colistin
Kp_HUCA_Bac_56	*bla*$_{CTX-M-15}$, *bla*$_{OXA-1}$, *bla*$_{OXA-48}$, *bla*$_{SHV-76}$, *bla*$_{TEM-1B}$, *aac(6′)-Ib-cr, oqxA, oqxB, qnrB1, fosA, aac(3)-IIa, aph(3′′)-Ib, aph(6)-Id, sul2, dfrA14, catB3, tet(A)*	AMP, AMC, P/T, CTX, FEP, ERT, CIP, SXT, GM, TB,	Meropenem + Amikacin
Kp_HUCA_Bac_57	*bla*$_{OXA-48}$, *oqxA, oqxB, fosA6, fosA7*	AMP, AMC, P/T, ERT	Ceftazidime
Kp_HUCA_Bac_59	*bla*$_{OXA-48,}$ *bla*$_{TEM-1A}$, *bla*$_{LEN-12}$, *oqxA, oqxB, fosA*	AMP, AMC, P/T, ERT	Ciprofloxacin
Kp_HUCA_Bac_60	*bla*$_{CTX-M-15}$, *bla*$_{OXA-1}$, *bla*$_{OXA-48,}$ *bla*$_{OXA-9}$, *bla*$_{SHV-28,}$	AMP, AMC, P/T, CTX,	Meropenem + Colistin

	bla$_{TEM-1A}$, *oqxA, oqxB, fosA6, aac(3)-IIa, aac(6′)-Ib3, aadA1, dfrA14, catB3, tet(D)*	FEP, ERT, CIP, SXT, GM, TB	
Kp_HUCA_Bac_62	*bla*$_{CTX-M-15}$, *bla*$_{OXA-1}$, *bla*$_{OXA-48}$, *bla*$_{SHV-76}$, *bla*$_{TEM-1B}$, *aac(6′)-Ib-cr, oqxA, oqxB, qnrB1, fosA, aac(3)-IIa, aph(3′′)-Ib, aph(6)-Id, sul2, dfrA14, catB3, tet(A)*	AMP, AMC, P/T, CTX, FEP, ERT, CIP, SXT, GM, TB	Piperacillin/Tazobactam
Kp_HUCA_Bac_63	*bla*$_{OXA-48}$, *bla*$_{SHV-12}$, *bla*$_{TEM-1B}$, *oqxA, oqxB, qnrB1, fosA, aac(3)-IId, aph(3′′)-Ib, aph(6)-Id, sul2, dfrA14, catA2, tet(A), tet(D)*	AMP, AMC, P/T, CTX, FEP, ERT, CIP, SXT, GM	Imipenem + Tobramycin
Kp_HUCA_Bac_64	*bla*$_{CTX-M-15}$, *bla*$_{OXA-1}$, *bla*$_{OXA-48}$, *bla*$_{SHV-1}$, *aac(6′)-Ib-cr, oqxA, oqxB, fosA5, aadA2, aph(3′)-Ia, sul1, mph(A), dfrA12, catB3*	AMP, AMC, P/T, CTX, FEP, ERT, IMP, MER, CIP, SXT	Gentamicin + Colistin + Tigecycline
Kp_HUCA_Bac_65	*bla*$_{OXA-48}$, *bla*$_{SHV-12}$, *bla*$_{TEM-1B}$, *oqxA, oqxB, qnrB1, qnrS1, fosA, aac(3)-IId, aph(3′′)-Ib, aph(6)-Id, sul2, dfrA14, catA2, tet(A), tet(D)*	AMP, AMC, P/T, CTX, FEP, ERT, IMP, MER, CIP, SXT, GM, FF	Meropenem + Amikacin
Kp_HUCA_Bac_66	*bla*$_{OXA-48}$, *bla*$_{SHV-12}$, *bla*$_{TEM-1B}$, *oqxA, oqxB, qnrB1, qnrS1, fosA, aac(3)-IId, aph(3′′)-Ib, aph(6)-Id, sul2, dfrA14, catA2, tet(D)*	AMP, AMC, P/T, CTX, FEP, ERT, CIP, SXT, GM	Meropenem + Amikacin
Kp_HUCA_Bac_67	*bla*$_{OXA-48}$, *bla*$_{SHV-12}$, *bla*$_{TEM-1B}$, *oqxA, oqxB, qnrB1, qnrS1, fosA, aac(3)-IId, aph(3′′)-Ib, aph(6)-Id, sul2, dfrA14, catA2, tet(A), tet(D)*	AMP, AMC, P/T, CTX, FEP, ERT, CIP, SXT, GM	Amikacin + Colistin
Kp_HUCA_Bac_68	*bla*$_{OXA-48}$, *bla*$_{SHV-27}$, *oqxA, oqxB, fosA6*	AMP, AMC, P/T, ERT	Cefotaxime + Ciprofloxacin
Kp_HUCA_Bac_69	*bla*$_{CTX-M-15}$, *bla*$_{OXA-1}$, *bla*$_{OXA-48}$, *bla*$_{OXA-9}$, *bla*$_{SHV-28}$, *bla*$_{TEM-1A}$, *aac(6′)-Ib-cr, oqxA, oqxB, fosA6, aac(3)-IIa, aac(6′)-Ib3, aadA1, dfrA14, catB3*	AMP, AMC, P/T, CTX, FEP, ERT, CIP, GM, TB	Meropenem + Colistin + Amikacin
Kp_HUCA_Bac_71	*bla*$_{OXA-48}$, *bla*$_{SHV-12}$, *bla*$_{TEM-1B}$, *oqxA, oqxB, qnrB1, fosA, aac(3)-IId, aph(3′′)-Ib, aph(6)-Id, sul2, dfrA14, catA2, tet(A), tet(D)*	AMP, AMC, P/T, CTX, FEP, ERT, CIP, SXT, GM, TIG	Meropenem + Amikacin + Colistin
Kp_HUCA_Bac_72	*bla*$_{CTX-M-15}$, *bla*$_{OXA-1}$, *bla*$_{OXA-48}$, *bla*$_{SHV-28}$, *bla*$_{TEM-1B}$, *aac(6′)-Ib-cr, oqxA, oqxB, qnrB1, fosA6, aph(3′′)-Ib, aph(6)-Id, aph(3′)-Ia, aph(4)-Ia, aac(3)-IV, sul2, dfrA14, catB3, tet(A)*	AMP, AMC, P/T, CTX, FEP, ERT, CIP, SXT, TB, FF, TIG	Meropenem + Amikacin
Kp_HUCA_Bac_73	*bla*$_{OXA-1}$, *bla*$_{OXA-48}$, *bla*$_{SHV-28}$, *bla*$_{TEM-1C}$, *aac(6′)-Ib-cr, oqxA, oqxB, fosA6, aac(3)-IId, catB3*	AMP, AMC, P/T, ERT, CIP, GM, TB, CST	Ceftazidime
Kp_HUCA_Bac_74	*bla*$_{CTX-M-15}$, *bla*$_{OXA-1}$, *bla*$_{OXA-48}$, *bla*$_{SHV-76}$, *bla*$_{TEM-1B}$, *aac(6′)-Ib-cr, oqxA, oqxB, qnrB1, fosA, aac(3)-IIa, aph(3′′)-Ib, aph(6)-Id, sul2, dfrA14, catB3, tet(A)*	AMP, AMC, P/T, CTX, FEP, ERT, CIP, SXT, GM, TB,	Amikacin
Kp_HUCA_Bac_75	*bla*$_{CTX-M-15}$, *bla*$_{OXA-1}$, *bla*$_{OXA-48}$, *bla*$_{SHV-28}$,	AMP, AMC, P/T, CTX,	Meropenem +

	bla_{TEM-1B}, *aac(6′)-Ib-cr, oqxA, oqxB, qnrB1, fosA6, aph(3′′)-Ib, aph(6)-Id, sul2, dfrA14, catB3, tet(A)*	FEP, ERT, CIP, SXT, TB, FF, CST	Tigecycline + Colistin
Kp_HUCA_Bac_76	$bla_{CTX-M-15}$, bla_{OXA-1}, bla_{OXA-48}, bla_{SHV-76}, bla_{TEM-1B}, *aac(6′)-Ib-cr, oqxA, oqxB, qnrB1, fosA, aac(3)-IIa, aph(3′′)-Ib, aph(6)-Id, sul2, dfrA14, catB3, tet(A)*	AMP, AMC, P/T, FOX, CTX, FEP, ERT, IMP, MER, CIP, SXT, GM, TB, FF	Meropenem
Kp_HUCA_Bac_78	bla_{OXA-48}, bla_{SHV-12}, bla_{TEM-1B}, *oqxA, oqxB, qnrB1, fosA, aac(3)-IId, aph(3′′)-Ib, aph(6)-Id, sul2, dfrA14, catA2, tet(A), tet(D)*	AMP, AMC, P/T, CTX, FEP, ERT, IMP, CIP, SXT, GM	Meropenem + Colistin
Kp_HUCA_Bac_79	bla_{OXA-48}, bla_{SHV-12}, bla_{TEM-1B}, *oqxA, oqxB, qnrB1, fosA, aac(3)-IId, aph(3′′)-Ib, aph(6)-Id, sul2, dfrA14, catA2, tet(A), tet(D)*	AMP, AMC, P/T, CTX, FEP, ERT, CIP, SXT, GM	Imipenem + Colistin
Kp_HUCA_Bac_80	$bla_{CTX-M-15}$, bla_{OXA-1}, bla_{OXA-48}, bla_{OXA-9}, bla_{SHV-28}, bla_{TEM-1A}, *aac(6′)-Ib-cr, oqxA, oqxB, fosA6, aac(6′)-Ib3, aadA1, dfrA14, catB3*	AMP, AMC, P/T, CTX, FEP, ERT, CIP, TB, FF	Meropenem
Kp_HUCA_Bac_81	$bla_{CTX-M-15}$, bla_{OXA-1}, bla_{OXA-48}, bla_{SHV-28}, bla_{TEM-1B}, *aac(6′)-Ib-cr, oqxA, oqxB, qnrB1, fosA6, aph(3′′)-Ib, aph(6)-Id, sul2, dfrA14, catB3, tet(A)*	AMP, AMC, P/T, CTX, FEP, ERT, CIP, SXT, FF, CST	Meropenem + Colistin
Kp_HUCA_Bac_82	bla_{OXA-48}, *oqxA, oqxB, fosA, aadA2b, sul1, tet(A)*	AMP, AMC, P/T, ERT, CIP	Cefotaxime + Amikacin + Colistin
Kp_HUCA_Bac_83	bla_{OXA-48}, bla_{SHV-12}, bla_{TEM-1B}, *oqxA, oqxB, qnrB1, fosA, aph(3′′)-Ib, aph(6)-Id, sul2, dfrA14, catA2, tet(A), tet(D)*	AMP, AMC, P/T, CTX, FEP, ERT, CIP, SXT	Meropenem + Amikacin + Tigecycline
Kp_HUCA_Bac_84	bla_{OXA-48}, bla_{SHV-11}, *oqxA, oqxB, fosA6*	AMP, AMC, P/T, ERT	Ciprofloxacin
Kp_HUCA_Bac_85	bla_{OXA-48}, $bla_{TEM-135}$, *oqxA, oqxB, qnrS1, fosA, dfrA14*	AMP, AMC, P/T, FOX, CTX, FEP, ERT, IMP, MER, CIP, SXT, FF	Tigecycline + Colistin
Kp_HUCA_Bac_88	bla_{OXA-48}, bla_{SHV-76}, *oqxA, oqxB, qnrB1, fosA, dfrA14*	AMP, AMC, P/T, ERT, IMP, MER, SXT, FF	Cefepime + Amikacin
Kp_HUCA_Bac_89	bla_{OXA-48}, bla_{SHV-12}, bla_{TEM-1B}, *oqxA, oqxB, qnrB1, fosA, aac(3)-IId, aph(3′′)-Ib, aph(6)-Id, sul2, dfrA14, catA2, tet(A), tet(D)*	AMP, AMC, P/T, CTX, FEP, ERT, CIP, SXT, GM	Tobramycin
Kp_HUCA_Bac_90	$bla_{CTX-M-15}$, bla_{OXA-1}, bla_{OXA-48}, bla_{SHV-1}, bla_{TEM-1A}, *aac(6′)-Ib-cr, oqxA, oqxB, fosA, aac(3)-IIa, dfrA14, catB3, tet(D)*	AMP, AMC, P/T, FOX, CTX, FEP, ERT, IMP, MER, CIP, GM, TB, CST	Colistin + Amikacin
Kp_HUCA_Bac_92	bla_{OXA-48}, bla_{SHV-12}, bla_{TEM-1B}, *oqxA, oqxB, qnrB1, fosA, aac(3)-IId, aph(3′′)-Ib, aph(6)-Id, sul2, dfrA14, catA2, tet(D)*	AMP, AMC, P/T, FOX, CTX, FEP, ERT, IMP, CIP, SXT, GM	Meropenem + Amikacin
Kp_HUCA_Bac_93	$bla_{CTX-M-15}$, bla_{OXA-1}, bla_{OXA-48}, bla_{SHV-28}, bla_{TEM-1B}, *aac(6′)-Ib-cr, oqxA, oqxB, qnrS1,*	AMP, AMC, P/T, CTX, FEP, ERT, CIP, SXT, GM,	Amikacin

	fosA6, aac(3)-IIa, aph(3'')-Ib, aph(6)-Id, sul2, dfrA14, catB3	TB, CST	
Kp_HUCA_Bac_94	*bla*$_{OXA-48}$, *bla*$_{SHV-12}$, *bla*$_{TEM-1B}$, *oqxA, oqxB, qnrB1, fosA, aac(3)-IId, aph(3'')-Ib, aph(6)-Id, sul2, dfrA14, tet(D)*	AMP, AMC, P/T, CTX, FEP, ERT, CIP, SXT, GM	Meropenem + Amikacin
Kp_HUCA_Bac_95	*bla*$_{OXA-48}$, *bla*$_{SHV-12}$, *bla*$_{TEM-1B}$, *oqxA, oqxB, qnrB1, qnrS1, fosA, aac(3)-IId, aph(3'')-Ib, aph(6)-Id, sul2, dfrA14, catA2, tet(D)*	AMP, AMC, P/T, CTX, FEP, ERT, CIP, SXT, GM	Meropenem + Amikacin

ND, not detected; AMP, ampicillin; AMC, amoxicillin/clavulanic acid; P/T, piperacillin/tazobactam; FOX, cefoxitin; CTX, cefotaxime; FEP, cefepime; ERT, ertapenem; IMP, imipenem; MER, meropenem; CIP, ciprofloxacin; SXT, trimethoprim-sulphamethoxazole; GM, gentamicin; TB, tobramycin; AK, amikacin; FF, fosfomycin; TIG, tigecycline; CST, colistin.
